# Electroacupuncture Alleviates Bladder Overactivity via Inhabiting Bladder P2X_3_ Receptor

**DOI:** 10.1155/2020/4080891

**Published:** 2020-03-16

**Authors:** Qi-fan Feng, An-dong Zhang, Man Xing, Xi Wang, Shu-ren Ming, Yue-lai Chen

**Affiliations:** ^1^Hainan Traditional Chinese Medicine Hospital, Haikou, Hainan 570203, China; ^2^Yueyang Hospital of Integrated Traditional Chinese and Western Medicine, Shanghai University of Chinese Medicine, Shanghai 200437, China

## Abstract

Electroacupuncture (EA) has been widely applied for overactive bladder, but the mechanism of its action remains to be clarified. This study was aimed to investigate EA regulating the effect of purinergic signaling in the OAB of rats. Electroacupuncture (continuous wave, 30 Hz, 1 mA) was applied to stimulate the Ciliao point (BL32) and the Huiyang point (BL35) of rats. Results showed that when the P2X_3_ receptor in bladder peripheral level and the spinal cord central level was involved in the bladder micturition reflex of the afferent signaling, intravenous administration P2X_3_ antagonist AF-353 can significantly inhibit urination in naive rats and OAB of rats and increase bladder volume and micturition pressure. EA stimulation alleviated bladder overactivity significantly and after the P2X_3_ receptor was blocked, the EA effect was weakened. EA stimulation can effectively reduce the P2X_3_ mRNA and protein expression in OAB of rats, spinal cord (L6-S1), and DRG (L6-S1) and can significantly reduce the number of positive P2X_3_ cells in OAB of rats, spinal cord (L6-S1), and DRG (L6-S1). These findings suggest that EA stimulation could alleviate bladder overactivity, and the function is closely related to the inhabited P2X_3_ receptor in the bladder.

## 1. Introduction

Overactive bladder (OAB) is defined as a symptom complex comprising urinary urgency, with or without urgency incontinence, usually with frequency and nocturia, in the absence of other local or metabolic factors that would account for the symptoms [1]. Many studies have proved that electroacupuncture stimulation could alleviate bladder overactivity, improve urine dynamics condition, and increase the bladder capacity [2–4]. The effect of acupuncture on bladder activity has been confirmed, but the underlying mechanisms are incompletely understood.

Purinergic signaling is an important contributor to normal urine storage and voiding and further implicated in bladder functional disorders related to interstitial cystitis/painful bladder syndrome, neurogenic bladder secondary to spinal cord injury, lower urinary tract symptoms, bladder outlet obstruction, diabetes, and aging [5–11]. In the bladder, P2X receptors act on the detrusor muscle in a motor role, whereas P2X_3_ receptors are thought to have a sensory role. This sensory function comprises both a physiological action in normal bladder filling and emptying cycles and a nociceptive action in pathological states. It has been shown that in bladder sensory neurons, P2X_3_ receptors are expressed and function as homomeric or heteromeric (P2X2/3) channels, as indicated by the use of selective antagonists [12, 13]. There was evidence of increased P2X_3_ expression in the urothelium of the OAB group [14]. The role of the P2X_3_ receptors in urgency and the pathophysiology of OAB has become the focus of intense interest.

Therefore, the present study was performed in rats to examine the following: (1) the effects of the P2X_3_ receptor in the bladder peripheral system contributor to bladder physiological action and pathological overactivity; (2) whether electroacupuncture on bladder overactivity is related to the P2X_3_ receptor mediation pathway.

## 2. Materials and Methods

### 2.1. Animals and Study Design

All experiments were performed on 40 female Sprague Dawley rats weighing 200–300 g, obtained from the Experimental Animal Center, Shanghai University of Traditional Chinese Medicine. The rats were maintained under standard laboratory conditions with a 12-h light/12-h dark cycle and free access to food pellets and tap water. The room temperature was maintained at 22 ± 1°C and relative humidity at 45% to 50%. All rats were acclimatized for at least 3 days before the experiments. All experiments were performed complying with international ethical standards in the care and use of animals and were approved by the Institutional Animal Care Committee of Shanghai University of Traditional Chinese Medicine.

### 2.2. Acupuncture Treatment

We followed the methods of Feng et al. [15], in which conscious rats were treated by acupuncture. One acupuncture needle (diameter, 0.22 mm) was positioned almost vertically underneath the periosteum about 5 mm lateral to the midline of the S_2_ (BL32, Ciliao), and the other acupuncture needle (diameter, 0.22 mm) was positioned almost vertically underneath the periosteum about 5 mm lateral to the midline of the coccyx (BL35, Huiyang), bilateral symmetry. It was stimulated electrically at both right and left points with a frequency of 30 Hz and intensity of 1 mA. The experiment was divided into in vivo experiment and in vitro experiment. In in vivo experiment, acupuncture was performed for 1 minute each time, and in in vitro experiment, acupuncture was performed for 3 days.

### 2.3. CYP-Induced OAB Operation and Cystometry

Rats were induced by intraperitoneal injecting with CYP (Sigma-Aldrich) (150 mg/kg). Cystometry was performed under 48 h after CYP injection, and rats were anesthetized by intraperitoneal injection of 20% urethane (0.5 ml/100 g). One end of the polyethylene catheter (diameter 0.42 mm) was inserted into the lumen through a small incision at the dome of the bladder for infusion of saline (0.9% NaCl, 0.2 ml/min), and the other was connected to a pressure transducer for recording intravesicular pressure. Another catheter was inserted into the right femoral artery and femoral vein, respectively, for recording the blood pressure and drug administration.

### 2.4. Hematoxylin-Eosin Staining

Bladder samples were fixed with 4% paraformaldehyde overnight and stained with hematoxylin and eosin as previously described [16].

### 2.5. RNA Extraction and Quantitative RT-PCR

The rats were euthanized under anesthesia of 20% urethane (0.5 ml/100 g), and then their abdomen were opened and urinary bladder were aseptically isolated and immediately frozen in liquid nitrogen and placed immediately to store at −80°C until use. Each sample was collected in 0.5 mL ice-cold Trizol (Invitrogen, Carlsbad, CA, USA). After its homogenization and sonification, RNA was extracted with chloroform, precipitated with isopropyl alcohol, and dissolved in 40 *μ*l RNAse-free DEPC. The RNA concentration and purity were analyzed by a Nanodrop spectrophotometer (Nanodrop Technologies, Wilmington, DE), with the spectral absorption at 260 and 280 nm. For cDNA synthesis, oligo (dT) primers, 1 *μ*g of each total RNA sample, and the RevertAid™ First Strand cDNA Synthesis Kit (Fermentas, Ontario, Canada) was used, following the guidelines of the manufacturer. cDNA samples were placed on ice and stored at −20°C until further use. Prior to the analysis, 10 *μ*l of each cDNA sample was diluted with 90 *μ*l of MilliQ water.

qPCRs were performed with the Prism 7900 Sequence Detection System (Applied Biosystems, Foster City, CA). Quantitative RT-PCR was carried out in 20 *μ*L buffer solution containing 1 *μ*L of diluted cDNA sample, 10 *μ*L 2 × SYBR green II qRT-PCR kit (Toyobo, Osaka, Japan), 1 *μ*L of each primer (5 *μ*M), and 8 *μ*l of MilliQ water. The primer sequences are listed in Table 1. Cycling conditions were 10 min 95°C, followed by 40 cycles of 15 sec at 95°C and 1 min at 60°C. After cycling, a melting protocol was performed with 15 sec at 95°C, 1 min at 60°C, and 15 sec at 95°C, to control for product specificity. The fold change (FC) in target gene cDNA relative to the selected endogenous control gene was determined as follows: FC = 2 − ΔΔCt, where ΔΔCt = (CtTarget − CtControl) test − (CtTarget − CtControl) control. Ct values were defined as the number of the PCR cycles at which the fluorescence signals were detected.

### 2.6. Western Blotting

Total protein was extracted from the frozen tissues, and protein concentration was quantified using the BCA protein assay kit (Beyotime Biotechnology Co., Haimen, Jiangsu, China). Samples (30 *μ*g total protein per loading) were separated on a 10% sodium dodecyl sulfate-polyacrylamide gel electrophoresis gel and electrotransfered onto polyvinylidene difluoride membranes (Millipore, Bedford, MA, USA). The membranes were blocked with 5% nonfat milk for 1 h at room temperature and then incubated with the primary antibodies recognizing *β*-actin (mouse monoclonal, 1: 5000, Abcam, Cambridge, UK) and P2X_3_ (rabbit polyclonal, 1 : 1000, Abcam, Cambridge, UK) at 4°C overnight. Then, the membranes were incubated with a horseradish peroxidase-conjugated anti-mouse (1: 5000, Abcam, Cambridge, UK) or goat anti-rabbit HRP (1: 4000, Abcam, Cambridge, UK). The signal was visualized with the ECL Plus reagent (GE healthcare, Buckinghamshire, UK) and exposed onto the X-ray film (Kodak, Rochester, NY, USA). Protein ratios were calculated based on densitometrical quantification of scanned films in Image J software.

### 2.7. Data Analysis

All data were expressed as mean ± SEM. Statistical significance was tested using one-way analysis of variance (ANOVA), with values of *P* < 0.05 considered significant.

## 3. Results

### 3.1. OAB Model Evaluation

**Figure 1:**
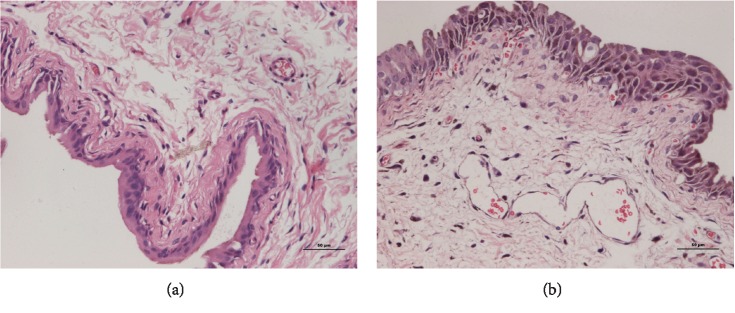
Observations of the bladder biopsy under a microscope (×200): cell nucleus was blue, and cytoplasm was red. Compared with naive rats, OAB rats bladder wall was thick, swollen, and hyperemia. Bladder epithelial cell exfoliation, tissue edema, and inflammatory cell infiltration were observed. (a) Naive rats. (b) OAB rats.

**Figure 2:**
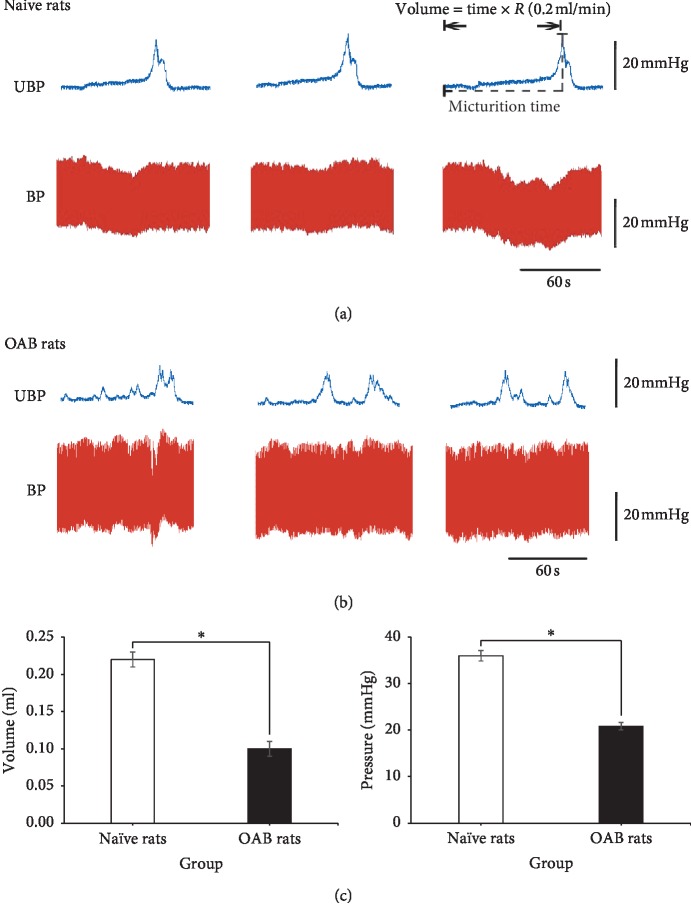
The original tracing of cystometrogram. (a, b) Naive rats and OAB rats original tracing of the cystometrogram. UBP: urine bladder pressure and BP: blood pressure. (c) Bladder volume and micturition pressure comparison between two groups. Bladder volume was micturition time × *R* (0.2 mL/min). *∗*Versus naive rats *P* < 0.05.

After 48 h of CYP intraperitoneal injection, the rat bladder wall was thick, swollen, and hyperemia. Under the microscope, we can observe the exfoliation of bladder epithelial cells, the edema of tissue, and the infiltration of inflammatory cells. Detrusor cells were larger, which showed a change in the vacuole model and the colour of the cytoplasm was deeper (Figure 1). In urodynamics, OAB rats bladder detrusor showed an unstable contraction wave. Compared with naive rats (0.22 ± 0.01 mL, *n* = 20), the bladder volume was significantly reduced in OAB rats (0.10 ± 0.01 mL, *n* = 20) (*P* < 0.001). Compared with naive rats (36.01 ± 1.10 mmHg, *n* = 20), the bladder micturition pressure was significantly decreased in OAB rats (20.84 ± 0.78 mmHg, *n* = 20) (*P* < 0.001). The OAB model was made successful (Figure 2).

### 3.2. Effects of P2X_3_ Antagonist AF-353 in Bladder Peripheral System on Bladder Activity

**Figure 3:**
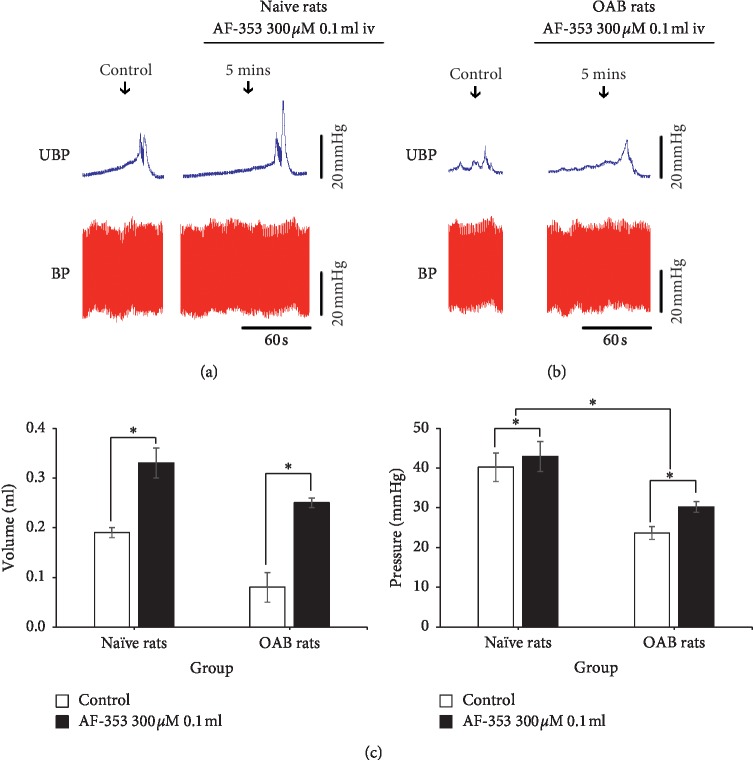
The original tracing of the cystometrogram after AF-353 (0.1 mL 300 *μ*M) intravenous administration. (a, b) Naive rats and OAB rats cystometrogram and blood pressure before and after intravenous administration. (c) Comparison of bladder volume and micturition pressure between two groups before and after intravenous administration. *∗*Versus control *P* < 0.05.

To evaluate P2X_3_ receptor effects of the bladder peripheral system on bladder activity, intravenous administration of AF-353 (0.1 mL 300 *μ*M) was applied. In comparison within the groups, in the naive rats (*n* = 4), after intravenous administration, the bladder volume (0.33 ± 0.03 mL) was significantly larger than before (0.19 ±0.01 mL) (*P* < 0.001) and the micturition pressure (42.90 ±3.75 mmHg) was significantly higher than before (40.22 ±3.59 mmHg) (*P* < 0.001). In OAB rats (*n* = 4), after intravenous administration, the bladder volume (0.25 ± 0.01 mL) was significantly larger than before (0.08 ± 0.03 mL) (*P* < 0.001) and the micturition pressure (30.25 ± 1.36 mmHg) was significantly higher than before (23.63 ± 1.60 mmHg) (*P* < 0.001). Comparing between the groups, before intravenous administration, compared with naive rats, OAB rats' bladder volume and micturition pressure obviously increased, respectively (*P* < 0.001). Before and after intravenous administration for difference comparison between groups, OAB rats micturition pressure ascending range (6.62 ± 1.35 mmHg) was significantly higher than naive rats (2.67 ± 0.55 mmHg) (*P*=0.017), but OAB rats' bladder volume rangeability (0.17 ± 0.01 mL) was no significantly different than naive rats (0.15 ± 0.03 ml) (*P*=0.507) (Figure 3).

### 3.3. Effects of EA Stimulation on Bladder Activity

**Figure 4:**
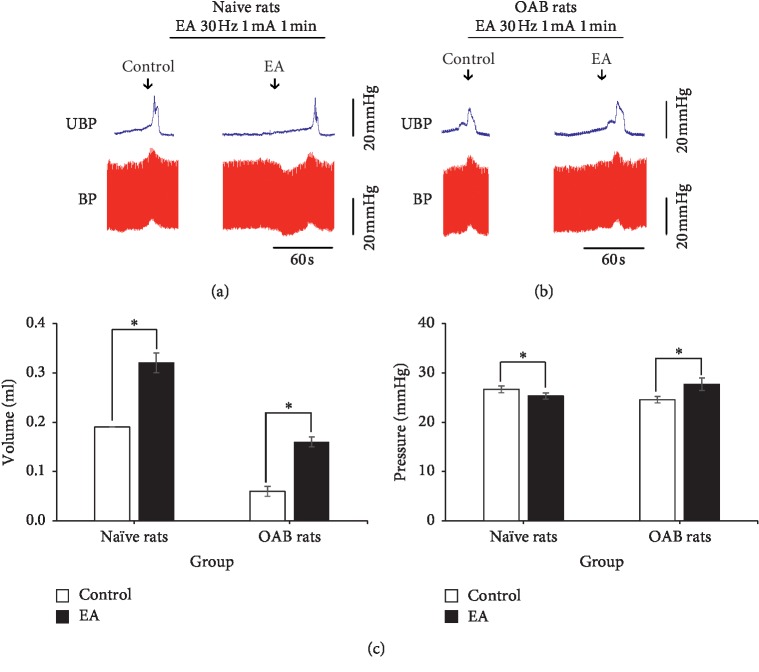
The original tracing of the cystometrogram after EA. (a, b) Naive rats and OAB rats cystometrogram and blood pressure before and after EA. (c) Comparison of bladder volume and micturition pressure between two groups before and after EA, ^*∗*^*P* < 0.05.

Comparing within the groups, in the naive rats (*n* = 4), after EA BL32 and BL35, the bladder volume (0.32 ± 0.02 mL) was significantly larger than before (0.19 ± 0.00 mL) (*P* < 0.001), but the micturition pressure (25.28 ± 0.63 mmHg) was not significantly different than before (26.64 ± 0.67 mmHg) (*P*=0.206). In OAB rats (*n* = 4), after EA, the bladder volume (0.16 ± 0.01 mL) was significantly larger than before (0.06 ± 0.01 mL) (*P* < 0.001) and the micturition pressure (27.69 ± 1.28 mmHg) was significantly higher than before (24.55 ± 0.63 mmHg) (*P* < 0.001). Comparing between groups, before EA, compared with naive rats, OAB rats' bladder volume and micturition pressure were obviously increased, respectively, (*P* < 0.001, *P*=0.037). Before and after EA for difference comparison between groups, OAB rats micturition pressure ascending range (3.14 + 0.75 mmHg) was significantly larger than naive rats (−1.35 + 1.03 mmHg) (*P*=0.003), but OAB rats' bladder volume (0.10 + 0.01 mL) was not significantly different in naive rats (0.13 + 0.02 mL) (*P*=0.305) (Figure 4).

### 3.4. Effects of AF-353 on EA Stimulation

**Figure 5:**
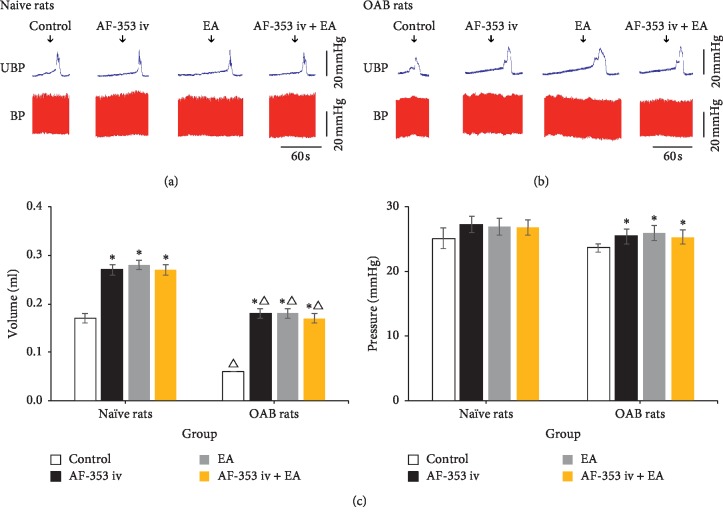
The original tracing of the cystometrogram after AF-353 iv and EA (30 Hz, 1 mA, 1 min). (a, b) Naive rats and OAB rats cystometrogram and blood pressure before and after AF-353 iv, EA, and AF-353 iv + EA. (c) Comparison of bladder volume and micturition pressure between two groups, ^*∗*^*P* < 0.05.

In the naive rats (*n* = 4), after intravenous administration AF-353 (0.1 mL 300 *μ*M), the bladder volume (0.27 ± 0.01 mL) was significantly large than before (0.17 ± 0.01 mL) (*P* < 0.001) and the micturition pressure (27.20 ± 1.25 mmHg) was no obviously different than the control (25.10 ± 1.63 mmHg) (*P*=0.212). When the bladder activity got back to the normal, EA was treated and the bladder volume (0.28 ± 0.01 mL) and the micturition pressure (26.88 ± 1.30 mmHg) were significantly increased compared with that of the control (*P* < 0.001). In EA treatment after intravenous administration, the bladder volume (0.27 ± 0.01 mL) was significantly increased compared with the baseline (*P* < 0.001) but was significantly decreased compared with only EA treatment (*P* < 0.001) and was no significantly different compared with only intravenous administration (*P*=0.06). The micturition pressure (26.80 ± 1.15 mmHg) was no significantly different compared with only intravenous administration and only EA treatment. In OAB rats (*n* = 4), after intravenous administration AF-353 (0.1 ml 300 *μ*M), the bladder volume (0.18 ± 0.01 mL) was significantly larger than before (0.06 ± 0.00 ml) (*P* < 0.001) and the micturition pressure (25.39 ± 1.16 mmHg) was significantly higher than before (23.61 ± 0.60 mmHg) (*P*=0.048). When the bladder activity got back to the normal, EA was treated and the bladder volume (0.18 ± 0.01 mL) and the micturition pressure (25.92 ± 1.17 mmHg) were significantly increased compared with the control (*P* < 0.001). In EA treatment after intravenous administration, the bladder volume (0.17 ± 0.01 mL) and the micturition pressure (25.27 ± 1.12 mmHg) were significantly increased compared with the control (*P* < 0.001, *P*=0.044), but there was no significant difference compared with only intravenous administration and only EA treatment (*P*=0.096*P*=0.268, *P*=0.617, *P*=0.051) (Figure 5).

### 3.5. Effects of EA Treatment on P2X_3_ Receptor

#### 3.5.1. Expression of P2X_3_ mRNA and Protein in Bladder

**Figure 6:**
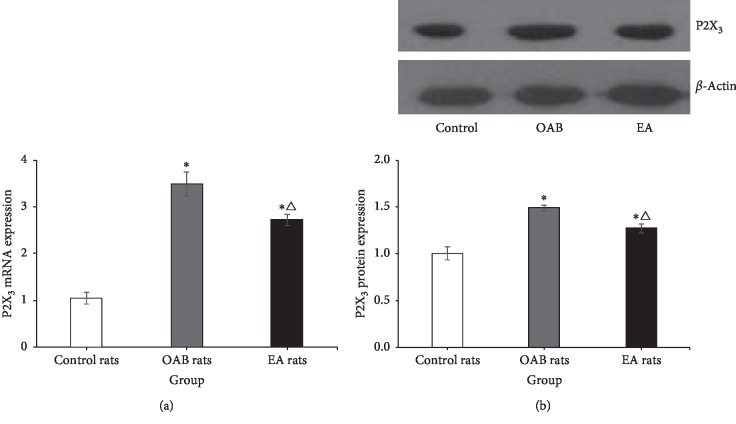
(a) Relative expression level of the P2X_3_ mRNA in the bladder. (b) Relative expression level of the P2X_3_ protein in the bladder.^*∗*^Versus the control rats group, *P* < 0.05. △Versus the OAB rats group, *P* < 0.05.

The expression of P2X_3_ mRNA in the bladder was significantly different among groups (*F* = 51.483, *P* < 0.001). The expression of P2X_3_ mRNA (3.49 ± 0.25, *n* = 6) in OAB rats was significantly higher than control rats (1.05 ± 0.13, *n* = 6) (*P* < 0.001) and EA rats (2.72 ± 0.11, *n* = 6) (*P*=0.006). The expression of P2X_3_ protein in the bladder was significantly different among groups (*F* = 20.233, *P* < 0.001). The expression of P2X_3_ protein (1.49 ± 0.03, *n* = 6) in OAB rats was significantly higher than control rats (1.00 ± 0.07, *n* = 6) (*P* < 0.001) and EA rats (1.27 ± 0.05, *n* = 6) (*P*=0.015) (Figure 6).

#### 3.5.2. Expression of P2X_3_ mRNA and Protein in Spinal Cord

**Figure 7:**
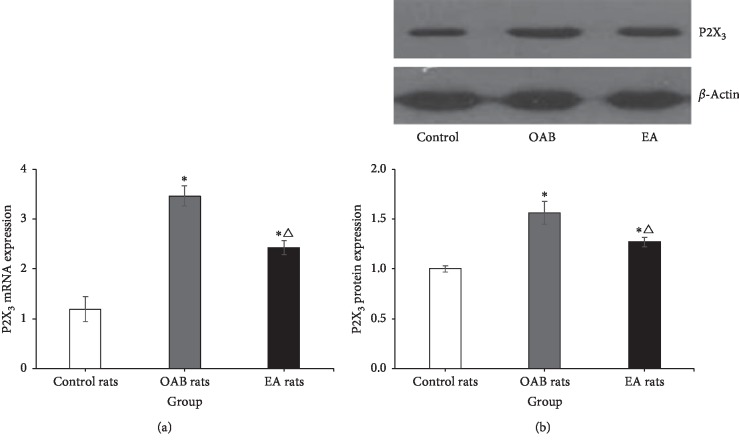
(a) Relative expression level of the P2X_3_ mRNA in the spinal cord. (b) Relative expression level of the P2X_3_ protein in the spinal cord.∗Versus the control rats group, *P* < 0.05. △Versus the OAB rats group, *P* < 0.05.

The expression of P2X_3_ mRNA in the spinal cord (L_6_-S_1_) was significantly different among groups (*F* = 30.514, *P* < 0.001). The expression of P2X_3_ mRNA (3.46 ± 0.20, *n* = 6) in OAB rats was significantly higher than control rats (1.19 ± 0.25, *n* = 6) (*P* < 0.001) and EA rats (2.42 ± 0.14, *n* = 6) (*P*=0.003). The expression of P2X_3_ protein in the spinal cord (L_6_-S_1_) was significantly different among groups (*F* = 16.026, *P* < 0.001). The expression of P2X_3_ protein (1.56 ± 0.11, *n* = 6) in OAB rats was significantly higher than control rats (1.00 ± 0.03, *n* = 6) (*P* < 0.001) and EA rats (1.27 ± 0.05, *n* = 6) (*P*=0.013) (Figure 7).

#### 3.5.3. Expression of P2X_3_ mRNA and Protein in DRG

**Figure 8:**
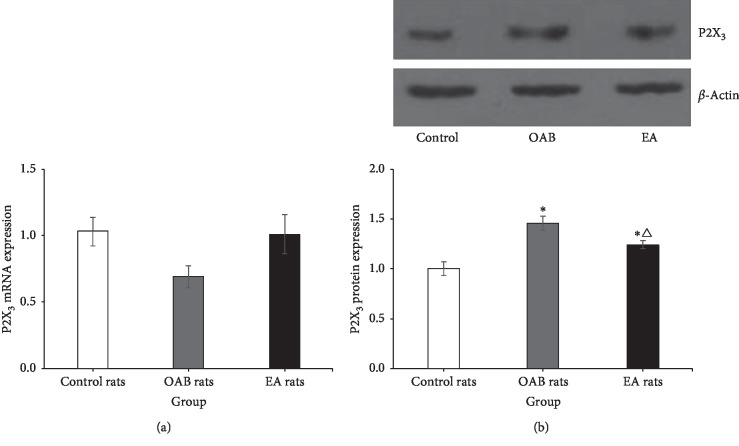
(a) Relative expression level of the P2X_3_ mRNA in DRG. (b) Relative expression level of the P2X_3_ protein in DRG.^*∗*^Versus the control rats group, *P* < 0.05. △Versus the OAB rats group, *P* < 0.05.

The expression of P2X_3_ mRNA in DRG (L_6_-S_1_) was no different among control rats (1.03 ± 0.11, *n* = 6), OAB rats (0.69 ± 0.08, *n* = 6), and EA rats ((1.01 ± 0.15, *n* = 6) (*F* = 2.741, *P*=0.097). The expression of P2X_3_ protein in DRG (L_6_-S_1_) was significantly different among groups (*F* = 15.023, *P* < 0.001). The expression of P2X_3_ protein (1.46 ± 0.07, *n* = 6) in OAB rats was significantly higher than control rats (1.00 ± 0.07, *n* = 6) (*P* < 0.001) and EA rats (1.24 ± 0.04, *n* = 6) (*P*=0.024) (Figure 8).

#### 3.5.4. Effects of EA Treatment on Distribution and Quantity of P2X_3_ Receptor

**Figure 9:**
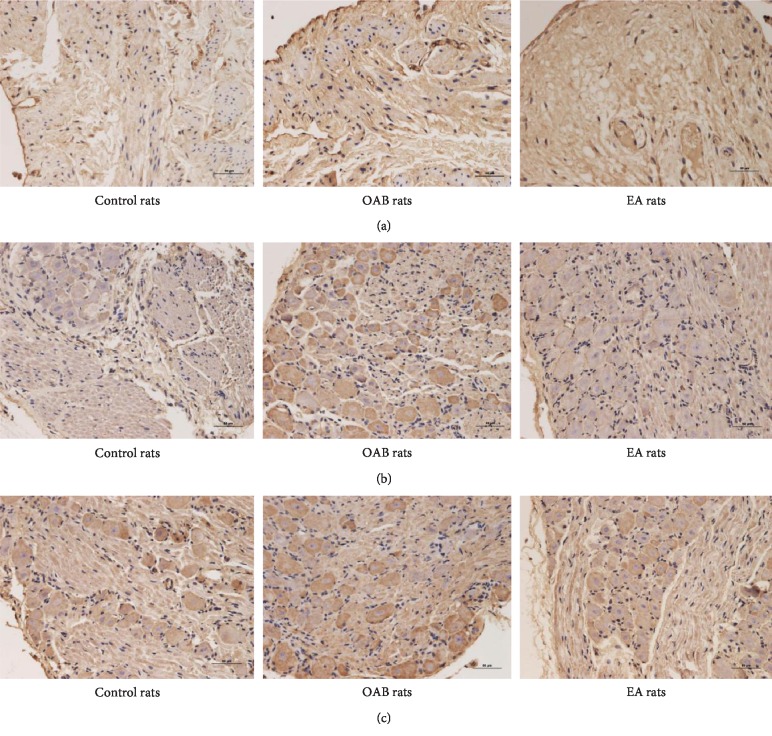
Immunohistochemical micrographs (×200) showing the distribution of the P2X_3_ receptor (brown). Nuclei (blue) were counterstained with DAPI. Scale bars represent 50 *μ*m in each case. (a) P2X_3_ receptor in the bladder; (b) P2X_3_ receptor in the spinal cord (L_6_-S_1_); (c) P2X_3_ receptor in DRG (L_6_-S_1_).

Immunohistochemical studies showed that P2X_3_ receptors were expressed in the rat bladder cytoplasm and cell membrane, detrusor muscle layer, nerve fiber bundles, and bladder transitional epithelial, especially in the cortex. In the L_6_-S_1_ spinal cord and DRG, P2X_3_ receptors were also expressed and highly expressed in a small diameter of DRG cells (Figure 9).

**Figure 10:**
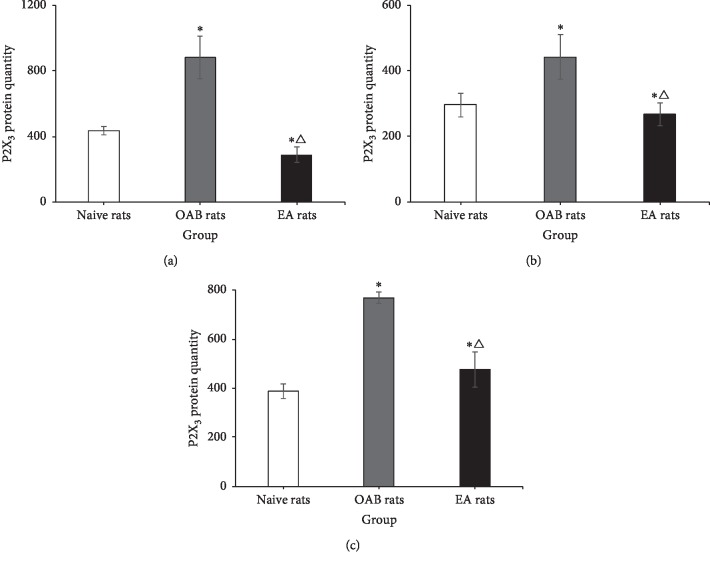
The quantity of the P2X_3_ receptor in the bladder, spinal cord, and DRG. ^*∗*^Versus the control rats group, *P* < 0.05. △Versus the OAB rats group, *P* < 0.05. (a) Bladder. (b) L_6_-S_1_ DRG. (c) (L6-S1) DRG.

P2X_3_ receptor in the bladder, spinal cord (L_6_-S_1_), and DRG (L_6_-S_1_) was counted in every visual field, and 10 visual fields were observed by random in every group. In the bladder, the results revealed a significant difference among these groups (*F* = 14.391, *P* < 0.001), and the quantity of the P2X_3_ receptor in OAB rats (883.80 ± 130.30, *n* = 4) was significantly increased compared with naive rats (436.10 ± 26.63, *n* = 4) (*P* < 0.001) and was less distributed in EA rats (287.60 ± 48.91, *n* = 4) (*P* < 0.001). In the spinal cord (L_6_-S_1_), there was a significant difference among these groups (*F* = 3.700 *P*=0.038), and the quantity of the P2X_3_ receptor in OAB rats (442.30 ± 68.06, *n* = 4) was significantly increased compared with naive rats (295.90 ± 36.50, *n* = 4) (*P*=0.043) and was less distributed in EA rats (267.40 ± 34.29, *n* = 4)(*P*=0.017). In DRG (L_6_-S_1_), there was a significant difference among these groups (*F* = 18.342, *P* < 0.001), and the quantity of the P2X_3_ receptor in OAB rats (769.40 ± 24.58, *n* = 4) was significantly increased compared with naive rats (388.30 ± 28.69, *n* = 4) (*P* < 0.001) and was less distributed in EA rats (475.60 ± 71.36, *n* = 4)(*P* < 0.001) (Figure 10).

## 4. Discussion

This study demonstrated that the P2X_3_ receptor which in bladder peripheral level was involved in the bladder micturition reflex of the afferent signaling, and intravenous administration P2X_3_ antagonist AF-353 can significantly inhibit naive rats and OAB rats bladder urination and increase bladder volume and micturition pressure. EA stimulation suppressed bladder overactivity significantly, and after the P2X_3_ receptor function was blocked by AF-353 intravenous administration, the EA effect was weakened.

Purinergic signaling is a term that relates to adenosine triphosphate (ATP) binding to its receptor (purinergic receptors such as P2X and P2Y subtypes). This pathway has been implicated in bladder functional disorders related to interstitial cystitis/painful bladder syndrome, neurogenic bladder secondary to spinal cord injury, lower urinary tract symptoms, diabetes, and aging. Purinergic signaling occurs at multiple sites, including the central nervous system, peripheral motor and sensory nerves, detrusor smooth muscle, and bladder urothelium [8]. Purinergic signaling is increasingly appreciated as an important contributor to the normal bladder functions of urine storage and voiding. The translation of disease and injury to abnormal sensory and motor function, resulting in lower urinary tract symptoms, is in part mediated by the interaction of adenosine 5′-triphosphate and related purines with specific purinergic receptors [17].

The role of purinergic signaling in the regulation of urinary bladder function was established when published reports demonstrated that P2X_2_/P2X_3_ knockout animals had significantly altered bladder function. P2X_3_ receptors are preferentially found on nerve fibres and are involved in bladder afferents signaling and pain transduction [18, 19]. There was evidence of increased P2X_3_ expression in the urothelium of the BOO group [20] locking the purinergic component which may be helpful in addition to antimuscarinics in blocking BOO-induced detrusor overactivity [21]. A study suggested that intravenous injection of the P2X_3_ antagonist AF-353 decreased the frequency of sensory field potentials evoked by activation of bladder noxious pathways and decreased the frequency of nonvoiding contractions in rats with neurogenic bladder hyperactivity [22]. Our studies were consistent with this. The results showed that intravenous administration of AF-353 could significantly inhibit naive rats and OAB rats' bladder urination and increase bladder volume and micturition pressure.

The acupuncture points of stimulation in this study were the classical points BL32 and BL35 corresponding to sacral vertebrae S 2 level and coccyx, which belongs to the Bladder Meridian of Foot Taiyang in traditional Chinese medicine. The clinical studies showed that acupuncture stimulation to the sacral vertebrae increases bladder capacity and suppresses overactive bladder [23, 24]. In our previous clinical studies, we found that electroacupuncture with continuous wave, 30 Hz, 1 mA stimulation could suppress bladder unstable contraction frequency and decrease detrusor maximum pressure in the bladder filling period thereby suppressing bladder overactivity. Therefore, we used the same therapy through the experiment.

In this study, we have found that EA stimulation alleviated bladder overactivity significantly, and after P2X_3_ receptor function was blocked by AF-353 intravenous administration, the EA effect was weakened. Immunohistochemical studies showed that P2X_3_ receptors were expressed in the rat bladder cytoplasm and cell membrane, the detrusor muscle layer, nerve fiber bundles, and bladder transitional epithelial, especially in the cortex, and the quantity of the P2X_3_ receptor in OAB rats was significantly increased compared with naive rats and was less distributed in EA rats. RT-PCR and western blotting studies showed that the expression of P2X_3_ mRNA and protein in OAB rats was significantly higher than naïve rats but could be reduced by EA treatment. These results implied that P2X_3_ receptors were closely associated with bladder sensory afferent, and EA regulation relied on P2X_3_ receptors mediated. EA treatment may influence bladder ATP release, lead to bladder cells P2X3 receptor function change and then change bladder sensory and motor functions and finally inhibit bladder overactivity. On the sacral spinal micturition center system, immunohistochemical studies showed that P2X_3_ receptors were expressed in L_6_-S_1_ spinal cord and DRG and highly expressed in a small diameter of DRG cells, and the quantity of the P2X_3_ receptor in OAB rats was significantly increased compared with naive rats and was less distributed in EA rats. RT-PCR and western blotting studies showed that the expression of P2X_3_ mRNA and protein in the L6-S1 spinal cord of OAB rats was significantly higher than naïve rats but could be reduced by EA treatment, and P2X_3_ protein expression in L_6_-S_1_ DAG of OAB rats was significantly higher than naive rats but could be reduced by EA treatment. These results implied that EA regulation relied on P2X_3_ receptors mediated in the sacral spinal micturition center system. The possible ways are (1) the EA signals may be uploaded to the brain central first and then descending inhibit ATP release of the presynaptic membrane, influencing the expression of P2X_3_ receptors in the spinal cord, reducing the bladder sensory signals transmission, finally inhibiting bladder overactivity. (2) EA signals directly affect the spinal dorsal horn neurons, influence the release of ATP, and lead to the combination change between ATP and P2X_3_ receptors on spinal cord dorsal horn neurons and then affect intracellular signaling pathways, finally inhibiting bladder overactivity.

Overactive bladder has a negative impact on quality of life. Electroacupuncture may potentially improve these symptoms. The present findings provide a scientific foundation to clinical treatment and point selection for the treatment of overactive bladder.

## 5. Conclusions

A systematic profile of the effects of EA effect on bladder overactivity and the P2X_3_ receptor mediation pathway was shown in the present study. The results show that P2X_3_ receptor which in bladder peripheral level and the spinal cord central level was involved in the bladder micturition reflex of the afferent signaling, blocking P2X_3_ receptor function which could inhibit bladder overactivity and EA stimulation which could significantly reduce the P2X_3_ protein expression in OAB rats bladder, spinal cord (L6-S1), and DRG (L6-S1). EA effect could alleviate bladder overactivity by the inhabited P2X_3_ receptor in the bladder.

## Figures and Tables

**Table 1:** Sequences of primers for real-time PCR.

Symbol	Forward primer	Reverse primer
Beta-actin	TCTGTGTGGATTGGTGGCTCT	AGAAGCATTTGCGGTGCAC
P2X_3_	AGGAGCCTCAGAGACCATCA	GGACATCTGGGCTATGGAGA

## Data Availability

All the individual participant data collected during the trial after publication will be available for anyone who wishes to access the data immediately following publication in accordance with principles.
